# Obstructive sleep apnea in psoriatic arthritis: A polysomnography-based Evaluation

**DOI:** 10.1097/MD.0000000000045699

**Published:** 2025-11-07

**Authors:** Mohammad Mustafa, Suzan Attar, Omar Kanbr, Yasser Bawazir, Ahmad A. Bamagoos, Ayah Boudal, Faris Alhejaili, Siraj Wali

**Affiliations:** a Rheumatology Unit, Department of Medicine, University of Jeddah, Jeddah, Saudi Arabia; b Rheumatology Division, Department of Medicine, King Abdulaziz University, Jeddah, Saudi Arabia; c College of Medicine, Umm Al-Qura University, Makkah, Saudi Arabia; d Sleep Medicine and Research Center, King Abdulaziz University Hospital, Jeddah, Saudi Arabia; e Department of Physiology, Faculty of Medicine in Rabigh, King Abdulaziz University, Rabigh, Saudi Arabia; f Rheumatology Unit, Department of Medicine, King Abdullah Medical Complex, Jeddah, Saudi Arabia; g Respiratory Division, Department of Medicine, King Abdulaziz University, Jeddah, Saudi Arabia.

**Keywords:** disease activity, obstructive sleep apnea, polysomnography, prevalence, psoriatic arthritis, rheumatology

## Abstract

Obstructive sleep apnea (OSA) is a challenging condition affecting 9% to 38% of people worldwide. However, its prevalence in patients with psoriatic arthritis (PsA) remains inconclusive. This study aimed to investigate the point-prevalence of OSA in patients with PsA using polysomnography (PSG) the gold-standard diagnostic method for OSA. Diagnosis of OSA was confirmed via standardized PSG utilizing the American Academy of sleep (AASM) recommendations. PsA disease activity was assessed using the disease activity index for psoriatic arthritis (DAPSA) and Health Assessment Questionnaire-Disability Index (HAQ-DI). The point-prevalence of OSA among the 42 PsA patients who underwent PSG was 50%, with 36% exhibiting moderate-to-severe OSA. No significant associations were identified between OSA occurrence and PsA disease activity assessed by DAPSA or HAQ-DI (*P* > .05). Older age (OR = 1.17, 95% CI = 1.07–1.27, *P* = .001) as well as greater BMI, which showed a trend toward significance (OR = 1.23, 95% CI = 0.99–1.52, *P* = .055), were predictors of OSA in patients with PsA. Although there was no correlation between PsA disease activity and OSA, our study highlights a higher prevalence of OSA among patients with PsA compared with the population in the same region, as well as reported literature.

## 1. Introduction

Psoriatic arthritis (PsA) is a member of the spondylarthritis group of diseases that are characterized by systemic autoimmune inflammations.^[1]^ Although the prevalence of PsA in the general population is estimated at 0.2%,^[2]^ the condition is associated with a devastating clinical picture.^[1,3]^ This includes peripheral arthritis, psoriasis, nail disease, axial involvement, dactylitis, enthesitis, and persistent fatigue.^[1]^ Additionally, patients with PsA often have metabolic syndrome, cognitive impairment, and poor quality of life.^[4–7]^ Comprehensive management and control of the condition are essential to improve patient daily functioning abilities and quality of life.^[3]^

Obstructive sleep apnea (OSA) is a sleep disordered breathing characterized by reduced oxygenation during sleep that can cause detrimental health sequelae if uncontrolled for long periods. These include metabolic syndrome, cardiovascular disease, neurocognitive impairment, and increased mortality.^[8]^ OSA affects at least one-third of people worldwide,^[9,10]^ and therapy is essential to improve patient daily functioning abilities, quality of life and prevent long term complications.^[10]^

Recently, a bidirectional relationship between OSA and PsA has been suggested. Egeberg et al reported patients with PsA were 75% more likely to have OSA compared to those without PsA, and patients with OSA were twice as likely to have PsA compared to non-OSA.^[11]^ Dejcman et al also reported that the prevalence of OSA among newly diagnosed patients with PsA is approximately 20%.^[12]^

Although the exact pathophysiology of PsA is unclear, overexpression of inflammatory cytokines is believed to play a key role.^[13]^ Indeed, the pathophysiological mechanism linking PsA with OSA may be explained by these inflammatory cytokines. Intermittent hypoxia and stress in patients with OSA can activate nucleic factor-kB, initiating an inflammatory cascade that leads to the release of tumor necrosis factor-alpha (TNF-α) and interleukin-6 (IL-6).^[5,14,15]^ Since these cytokines are implicated in the pathogenesis of PsA, therefore, early detection and targeted-intervention of OSA in patients with PsA seems important for improving diseases prognosis.^[16–19]^ However, not all studies show clear causal relationship between OSA and PsA. A Mendelian study investigated the relationship between OSA and all rheumatic diseases showed no direct causal relationship between OSA and PsA.^[20]^ Nonetheless, other Mendelian randomization studies showed that certain inflammatory proteins may increase the risk of OSA, while others appear protective.^[21,22]^

To this end, the relationship between OSA and PsA was based on few studies, most of which have not used objective measurements for OSA diagnosis, and none have employed polysomnography (PSG), the gold-standard tool for OSA diagnosis. Hence, the current study aimed to determine the prevalence of OSA in patients with PsA using the standardized diagnostic methodology of PSG. In addition, it also aimed to explore the clinical relationship between currently treated PsA and OSA.

## 2. Methods

### 2.1. Participants and protocol

Potential participants were all adult patients who met the CASPAR classification criteria for the diagnosis of PsA,^[23]^ attended the Rheumatology clinics at King Abdulaziz University Hospital, Jeddah, Saudi Arabia, between September 2023 and September 2024. Potential participants were provided with a detailed description of the study, and a written informed consent was signed by those willing to participate. This study was approved by the Ethics Committee of King Abdulaziz University Hospital, Jeddah, Saudi Arabia (Approval Number: 435-23). All methods were performed in accordance with the relevant guidelines and regulations, including the Declaration of Helsinki. Informed consent was obtained from all participants prior to their inclusion in the study.

During the clinic visit, a trained rheumatologist assessed participants’ current PsA disease activity using the disease activity index for psoriatic arthritis (DAPSA) and the Health Assessment Questionnaire-Disability Index (HAQ-DI). Detailed data on PsA therapy were systematically collected; however, information on inflammatory markers was not obtained due to the high cost of testing and resource constraints. Additionally, data on disease duration were not systematically available for all participants. Participants were then referred to the Sleep Clinics, and a trained sleep physician conducted a personal interview to collect demographic data, medical history, responses to the STOP-BANG questionnaire for OSA risk assessment, and Epworth Sleepiness Scale (ESS) scores for excessive daytime sleepiness (EDS) assessment. Anthropometric measurements, including height, weight, body mass index (BMI), and neck circumference were also obtained following standard clinical protocols. All agreeing participants underwent a PSG sleep study to determine the diagnosis and severity of OSA.

### 2.2. Instrumentation and procedures

#### 2.2.1. STOP-BANG questionnaire

The STOP-BANG questionnaire (snoring, tiredness, observed apnea, high BP, BMI, age, neck circumference, and gender) is a reliable, easy-to-use screening tool for the risk of OSA. It consists of 8 dichotomous (yes/no) items related to the clinical features of OSA. the total score ranges from 0 to 8, classifying patients into low risk of OSA (0–2), intermediate risk of OSA (3–4), or high risk of OSA (5–8). The questionnaire has a sensitivity of 93% for detecting moderate-to-severe OSA and up to 100% for detecting severe OSA.^[24]^

#### 2.2.2. Epworth Sleepiness Scale (ESS)

ESS is a validated questionnaire used to assess daytime sleepiness. It can reliably classify individuals as experiencing normal or EDS. Respondents rate their likelihood of dozing off in 8 daily situations using a 4-point Likert scale. The total score, ranging from 0 to 24, reflects the average propensity to fall asleep in these scenarios. A Score of 10 or less is considered normal, whereas a score >10 indicates EDS.^[25]^

#### 2.2.3. Disease activity index for psoriatic arthritis (DAPSA)

DAPSA is a scoring system developed based on recommendations from the Outcome Measures in Rheumatology (OMERACT) module. It assesses PsA disease activity across 5 clinical domains: a patient global assessment, pain assessment using a visual analog scale, a 68-tender joint count, a 66-swollen joint count, and CRP levels. Disease activity is categorized as follows: scores 0 to 4 indicate remission, 5 to 14 reflect low disease activity, 15 to 28 signify moderate activity, and scores above 28 indicate high disease activity.^[26,27]^

#### 2.2.4. Health Assessment Questionnaire-Disability Index (HAQ–DI)

The HAQ-DI is a functional status measure widely used across various diseases, including PsA.^[28]^ It evaluates 20 functions within 8 categories, such as dressing, eating, reaching, and walking over the preceding week. Each category is rated on a 4-point scale: 0 = without difficulty, 1 = with some difficulty, 2 = with much difficulty, and 3 = unable to do. The total score ranges from 0 to 3 in 0.125 increments, with higher scores indicating greater impairment. A score of 0 denotes no functional limitation, and a score of 3 indicates complete impairment.^[29]^

#### 2.2.5. Diagnostic polysomnography (PSG)

Participants underwent a standardized diagnostic PSG at our sleep center (Type 1) or at home (Type 2). The same device (SOMNO Medics Plus; SOMNO Medics, Randersacker, Germany) was used in both locations to ensure consistency of data. PSG recordings included electrocardiography, electroencephalography, electrooculography, electromyography (submental and bilateral anterior tibialis muscles), nasal pressure, nasal and oral airflow, chest and abdominal impedance belts for respiratory muscle effort, pulse oximetry, a tracheal microphone for snoring, and a body position sensor.

Sleep stages and respiratory events were manually scored by registered polysomnographic technologists using the 2020 American Academy of Sleep Medicine (AASM) scoring manual (Version 2.6).^[30]^ Apnea and hypopnea events per hour of sleep (apnea–hypopnea index, AHI), and oxygen desaturation of ≥ 3% per hour of sleep (oxygen desaturation index, ODI) were calculated.

OSA was diagnosed according to the most recent AASM criteria, which includes the following: 1) an AHI of ≥ 15 events/hour determined by PSG or 2) an AHI of ≥ 5 but < 15 events/hour, in addition to one of the following: daytime sleepiness, nonrestorative sleep, fatigue, or insomnia symptoms; waking up with gasping or choking sensations; and reported snoring, breathing interruption or both during sleep.^[31]^ Furthermore, OSA severity was classified based on AHI as mild OSA (AHI 5–<15 events/hour), moderate OSA (AHI 15–<30 events/hour), and severe OSA (AHI > 30 events/hour).^[31]^

### 2.3. Data analysis

Data was analyzed using SPSS software, version 26. The Shapiro–Wilk test was performed to assess data normality. The chi-square test (χ^2^) was performed to investigate the association between qualitative variables (expressed as numbers and/or percentages). An independent sample *t*-test was performed to assess differences in quantitative variables between OSA-positive and OSA-negative patients with PsA, with results expressed as mean and standard deviation (Mean ± SD). Binary logistic regression analysis was performed to identify the predictors of OSA in the study population. *P*-values < .05 was considered statistically significant.

## 3. Results

### 3.1. Participants’ general characteristics

Sixty-eight potential participants with PsA were initially offered enrollment into the current study. All potential participants were naïve to PSG and had never been diagnosed with OSA. Of these, 42 agreed to participate and undergo a diagnostic PSG. Participants (N = 42) were predominantly females (71%). Their average age was 47 ± 14 years, and their average BMI was 30 ± 5 kg/m^2^. The average ESS score was 7 ± 5, and 21% of participants had EDS (ESS score > 10). The average STOP-BANG score was 3 ± 2, and the proportions of participants having low, intermediate, or high risk of developing OSA were 33%, 36%, or 31%, respectively. Sleep-related symptoms were reported by 88% of patients, including snoring (71%), morning tiredness (79%), and witnessed apnea (26%). Additionally, 45% of patients had at least one comorbid condition, such as hypertension, diabetes, and dyslipidemia (see Table 1).

**Table 1:** Compares participants with and without OSA.

Variable (units)	Total (n = 42)	No OSA (n = 21)	OSA (n = 21)	*P*
Female gender (%n)	71%	81%	61%	.17
Age (yr)	47 ± 14	38.2 ± 10.8	55.8 ± 9.8	**<.01**
BMI (kg/m^2^)	30 ± 5	27.8 ± 6	31.5 ± 3.7	**.02**
ESS (score)	7 ± 5	5.6 ± 4.6	7.4 ± 5	.2
EDS (% n)	9 (21%)	3 (14%)	6 (29%)	.3
STOP-BANG (score)	3 ± 2	2.2 ± 1.3	4.4 ± 1.5	**<.01**
Risk of OSA (%n)				
Low	14 (33%)	13 (61%)	1 (5%)	**<.01**
Intermediate	15 (36%)	6 (29%)	9 (43%)	
High	13 (31%)	2 (10%)	11 (52%)	
Any OSA symptom	37 (88%)	16 (76%)	21 (100%)	**.017**
Snoring symptom	30 (71%)	10 (48%)	20 (95%)	**<.01**
Tiredness symptom	33 (79%)	15 (71%)	18 (86%)	.3
Witnessed apnea symptom	11 (26%)	4 (19%)	7 (33%)	.3
Any comorbidity	19 (45%)	10 (48%)	9 (43%)	.7
DAPSA (score)	18 ± 12	15.7 ± 8.8	20.8 ± 12.4	.3
DAPSA classification (%n)				
Remission to low disease	18 (43%)	9 (43%)	9 (43%)	1.0
Moderate to high disease	24 (57%)	12 (57%)	12 (57%)	
HAQ-ID (score)	0.7 ± 0.8	0.5 ± 0.7	0.8 ± 0.9	.2
HAQ-ID classification (%n)				
Low disability	30 (71%)	16 (76%)	14 (67%)	.5
Moderate to high disability	12 (29%)	5 (24%)	7 (33%)	
TST (min)	343 ± 91	360.1 ± 93	327 ± 89	.2
REM sleep time (%TST)	16 ± 8	14.5 ± 8.2	17.1 ± 7.8	.3
SE (%)	71 ± 17	73.9 ± 16.3	68.1 ± 18.3	.3
AHI (events/h)	13 ± 15	2.5 ± 1.8	23.3 ± 14.4	**<.01**
AHI REM (events/h)	25 ± 26	6.4 ± 7.7	43 ± 25.9	**<.01**
AHI Supine (events/h)	16 ± 19	3.7 ± 2.7	28.7 ± 19.6	**<.01**
MinO2 (%)	86 ± 7	87.9 ± 6.3	83.3 ± 7.5	.04
T90 (min)	2 ± 3	1.2 ± 3.5	1.9 ± 3.5	.5
ODI (events/h)	10 ± 9	2.8 ± 2.5	16.8 ± 8.6	**<.01**

Statistical analyses performed were independent *t*-tests for continuous variables and chi-square tests for categorical variables. Bold value indicates statistical significance (*P* < .05).

AHI = apnea–hypopnea index, BMI = body mass index, DAPSA = disease activity index for psoriatic arthritis (remission ≤ 4, low disease activity > 4 and ≤ 14, moderate disease activity > 14 and ≤ 28, high disease activity > 28), EDS = excessive daytime sleepiness, ESS = Epworth Sleepiness Scale (low risk of EDS: <10, high risk of EDS ≥ 10), HAQ-ID = Health Assessment Questionnaire-Disability Index (mild disability: 0–1, moderate disability:  > 1–2, severe disability: >2–3), MinO2 = minimum oxygen saturation, ODI = oxygen desaturation index, OSA = obstructive sleep apnea, PSG = polysomnography, REM = rapid eye movement, SE = sleep efficiency, STOP-BANG = snoring, tiredness, observed apnea, blood pressure, BMI, age, neck circumference, gender (low risk of OSA: 0–2, intermediate risk of OSA: 3–4, high risk of OSA: ≥5), T90 = sleep time spent with oxygen saturation below 90%, TST = total sleep time.

No significant difference was found between participants (N = 42) and those refusing to participate (N = 26) in the proportion of female participants (71% vs 65%, respectively, chi-square test, *P*-value = 0.6), age (47 ± 14 vs 45 ± 13 years, respectively, mean difference = -1.7, 95% CI = -8.1–4.9, independent *t*-test *P*-value = 0.6), BMI (30 ± 5 vs 29 ± 5, respectively, mean difference = −1.0, 95% CI = −3.6 to 1.5, independent *t*-test *P*-value = 0.4), ESS score (7 ± 5 vs 5 ± 5, respectively, mean difference = 1.6, 95% CI = 1.2–4.0, independent *t*-test *P*-value = 0.2), or the proportion of participants having EDS (21% vs 15%, respectively, chi-square test, *P*-value = 0.8).

### 3.2. Participants’ PsA disease activity

The average participants’ scores of DAPSA and HAQ-ID were 18 ± 12 and 0.7 ± 0.8, respectively. According to the DAPSA, the proportion of participants having remission, low, moderate, or high disease activity were 5%, 38%, 43%, or 14%, respectively. According to the HAQ-ID, the proportion of participants having mild, moderate, or severe functional disability were 71%, 21%, or 8%, respectively.

The proportion of female was higher in participants with active PsA (moderate-high DAPSA classification) compared to those with low PsA disease activity (remission to low DAPSA classification) (83% vs 56%, respectively, chi-square test, *P*-value = 0.049). However, no significant difference was observed between these 2 groups in terms of age (44 ± 13 years for patients with active PsA vs 51 ± 13 years for the remaining patients, mean difference = 7.1, 95% CI = −1.2 to 15.0, independent *t*-test, *P*-value = 0.1), BMI (31 + 5 vs 28 + 5, respectively, mean difference = −2.3, 95% CI = −5.6 to 0.9, independent *t*-test, *P*-value = 0.2), ESS score (7 ± 5 vs 6 ± 5, respectively, mean difference = 0.6, 95% CI = 1.5–2.5, independent *t*-test, *P*-value = 0.7), the proportion of participants having EDS (17% vs 28%, respectively, chi-square test *P*-value = 0.4), STOP-Bang score (3 ± 2 in both groups, mean difference = 0.0, 95% CI = −1.2 to 1.2, independent *t*-test *P*-value = 0.97) or the proportion of participants having moderate to high risk of developing OSA (63% vs 72%, chi-square test, *P*-value = 0.2). See Table 2.

**Table 2:** Compares between participants with active and inactive PsA disease.

Variable (units)	Moderate to high disease activity PSA (n = 24)	Remission to low disease activity PSA (n = 18)	*P*
DAPSA (score)	25.2 ± 9.1	8.9 ± 3.5	**>.01**
Female gender (%n)	83%	56%	**.049**
Age (yr)	44 ± 13	51 ± 13	.09
BMI (kg/m^2^)	31 ± 5	28 ± 5	.2
ESS (score)	6 ± 5	7 ± 5	.7
EDS (% n)	4 (17%)	5 (28%)	.4
STOP-BANG (score)	3 ± 2	3 ± 2	1.0
High risk of OSA (%n)	15 (63%)	13 (72%)	.2
HAQ-ID (score)	0.73 ± 0.87	0.59 ± 0.74	.6
Prevalence of OSA (%n)	12 (50%)	10 (56%)	.7
TST (min)	355 ± 71	328 ± 113	.3
REM sleep time (%TST)	16 ± 7	16 ± 9	.9
SE (%)	72 ± 17	70 ± 19	.8
AHI (events/h)	11 ± 13	15 ± 16	.4
AHI REM (events/h)	24 ± 27	25 ± 27	.9
AHI Supine (events/h)	14 ± 15	20 ± 23	.3
MinO2 (%)	85 ± 7	87 ± 7	.5
T90 (min)	1 ± 2	2 ± 5	.5
ODI (events/h)	9 ± 9	11 ± 11	.8

Statistical analyses performed were the independent *t*-test for continuous variables and chi-square test for categorical variables. Bold value indicates statistical significance (*P* < .05).

AHI = apnea–hypopnea index, BMI = body mass index, DAPSA = disease activity index for psoriatic arthritis (remission ≤ 4, low disease activity > 4 and ≤ 14, moderate disease activity > 14 and ≤ 28, high disease activity > 28), ESS = Epworth Sleepiness Scale (low risk of EDS: <10, high risk of EDS ≥ 10), EDS = excessive daytime sleepiness, HAQ-DI = Health Assessment Questionnaire-Disability Index (mild disability: 0–1, moderate disability: >1–2, severe disability: >2–3), MinO2 = minimum oxygen saturation, ODI = oxygen desaturation index. OSA = obstructive sleep apnea, PSG = polysomnography, REM = rapid eye movement, SE = sleep efficiency, STOP-BANG: (snoring, tiredness, observed apnea, blood pressure, BMI, age, neck circumference, gender) – (low risk of OSA: 0–2, intermediate risk of OSA: 3–4, high risk of OSA: ≥5), T90 = time spent below 90% oxygen saturation, TST = total sleep time.

No significant difference was found between participants (N = 42) and those refusing to participate (N = 26) in the DAPSA score (18.2 ± 10.9 vs 22.3 ± 17.4, respectively, mean difference = 4, 95% CI = −2.9 to 10.9, independent *t*-test *P*-value = 0.3) or the HAQ-ID score (0.67 ± 0.81 vs 0.58 ± 0.75, respectively, mean difference = −0.08, 95% CI = −0.48 to 0.30, independent *t*-test *P*-value = 0.7).

### 3.3. Prevalence of OSA in PsA

The PSG-based point-prevalence of OSA in participants with PsA (n = 42) was 50% (n = 21). Of these, 14% (n = 6) had mild OSA, 19% (n = 8) had moderate OSA, and 17% (n = 7) had severe OSA. The power of the study was found to be > 99% based on our sample size (n = 42), the standard alpha value (0.05), the OSA prevalence in the population of Saudi Arabia (8.8%),^[10]^ and the OSA prevalence in the current study (50%). Participants with OSA significantly differed from those without OSA in several factors, including age, BMI, STOP-BANG score, in addition to PSG parameters including AHI and ODI. Table 1 compares these characteristics between participants with and without OSA.

Snoring was significantly more prevalent among participants with OSA compared to those without (95% vs 48%, *P* < .01), affecting all patients in the mild and moderate OSA groups and 86% in the severe OSA group. Tiredness, witnessed apnea, or the presence of at least one of these symptoms was reported by 79%, 26%, and 88% of the total cohort, respectively, with no significant differences between OSA and non-OSA groups (*P* > .05). The frequency of comorbidities was also similar across groups (*P* = .9).

### 3.4. Relationship between OSA and PsA

We observed no difference between participants with OSA compared with those without OSA in DAPSA score (20.8 ± 12.4 vs 15.7 ± 8.8, respectively, mean difference = −5.0, 95% CI = −11.7 to 1.6, independent *t*-test *P*-value = 0.3), the proportion of participants having moderate-high DAPSA classification (43% for both groups, chi-square test *P*-value = 0.98), HAQ-ID score (0.8 ± 0.9 vs 0.5 ± 0.7 respectively, mean difference = −0.35, 95% CI = −0.85 to 0.15, independent *t*-test *P*-value = 0.2), or the proportion of participants having moderate-high HAQ-ID classification (67% vs 76%, respectively, chi-square test *P*-value = 0.5) (see Table 1).

No difference was also observed between participants with active PsA compared to those with inactive PsA in OSA prevalence or any other PSG measurements. The detailed comparison is presented in Table 2.

Biologic disease-modifying antirheumatic drugs (bDMARDs) were the most common prescribed therapy (n = 17, 40%) among our 42 participants. This proportion was higher among participants without OSA (n = 10, 50%) compared to participants with OSA (n = 7, 31.8%). Conventional disease-modifying antirheumatic drugs (csDMARDs) were used by 13 patients (31%), with similar distribution between OSA (n = 7, 31.8%) and No OSA groups (n = 6, 30%). Combination therapy was more frequent in patients with OSA (n = 5, 22.7%) compared to those without OSA (n = 2, 10%). Corticosteroid monotherapy was observed only in one participant from the No OSA group (n = 1, 2.3%). Patients not receiving treatment were slightly more common in the OSA group (n = 3, 13.6%) than in the No OSA group (n = 1, 5%).

### 3.5. Predictors of OSA

To identify possible predictors of OSA in participants within the PSG group, a binary logistic regression was also performed using a one-block forward selection method. The variables in the model were restricted to 4 only (i.e., DAPSA score, HAQ-DI score, BMI, and age) to reduce overfitting of the model. We selected these variables based on a combination of statistical and clinical considerations. Specifically, DAPSA is a validated composite measure of PsA disease activity and is widely recognized as an important clinical indicator of patient outcomes. HAQ-DI reflects physiological functionality which is strongly linked with PsA disease burden. Age and BMI are important demographic factors associated with both PsA and OSA disease severity. Multicollinearity assessment showed no significant violation of variance inflation factor values (maximum = 1.25) or tolerance values (minimum = 0.8). Two of the 4 variables were retained in the final logistic regression model (model x^2^ = 19.4, *P*-value < 0.001) as independent predictors of OSA in PsA: older age (OR = 1.17, 95% CI = 1.07–1.27, *P*-value = 0.001) and higher BMI (showed a trend toward significant) (OR = 1.23, 95% CI = 0.99–1.52, *P*-value = 0.055). We have also run a logistic regression using the “Enter” method and the metrics for BMI and Age were relatively consistent: age (OR = 1.2, 95% CI = 1.07–1.3, *P*-value = 0.001), BMI (OR = 1.2, 95% CI = 0.98–1.55, *P*-value = 0.07), DAPSA (OR = 1.09, 95% CI = 0.96–1.25, *P*-value = 0.19), HAQ (OR = 2.13, 95% CI = 0.4–11.0, *P*-value = 0.37).

## 4. Discussion

To our knowledge, this study was first to measure the frequency of OSA in patients with PsA using standardized PSG sleep studies. Fifty percent of patients with PsA had OSA, two-thirds of whom had moderate-to-severe disease. Age and BMI showed ability to predict OSA in patients with PsA. No significant associations were identified between OSA and PsA disease activity assessed by the DAPSA and HAQ-DI.

The prevalence of OSA among patients with PsA in the current study is relatively high, and is greater than that seen in the population of the same region^[9,10]^ and in previously reported similar studies,^[12,32]^ despite the limitation of sample size. Dejcman et al.^[12]^ investigated the prevalence of OSA in 16 newly diagnosed patients with PsA using polygraphy, reporting a 20% OSA prevalence (AHI ≥ 5), with 16.3% having moderate-to-severe OSA.^[12]^ Similarly, Petzinna et al^[32]^ conducted a case-control study that revealed a 29.4% (5/17) prevalence of OSA (AHI ≥ 5) in patients with PsA. They advocated incorporating routine screening tools, such as the ESS questionnaire and polygraphy, as accessible, cost-efficient diagnostic options for all newly diagnosed patients with PsA.^[32]^ This variance in the reported prevalence can be attributed to several factors including research methodologies. We used the gold-standard diagnostic tool, PSG sleep studies, to estimate OSA prevalence in patients with PsA. This provides a more precise and reliable measurement of AHI and, therefore, accurate identification of OSA severity compared to other diagnostic modalities. For example, polygraphy relies on the total recording time as a surrogate measure to actual sleep time for determining AHI. This often causes underestimation of OSA diseases diagnosis and severity.^[33]^ We also applied the latest, and more rigorous, recommended criteria from the AASM for OSA diagnosis (AHI ≥ 5 with any OSA symptom or AHI ≥ 15).^[31]^ This is unlike many previous reports that used the epidemiological definition to diagnose OSA (AHI ≥ 5). These methodological advantages strengthen the validity of our findings, but further validation of our results in future studies is still required. Collectively, however, these studies and ours suggest that physicians should always maintain a high level of suspicion regarding OSA when providing routine clinical care to patients with PsA.

Interestingly, all participants with OSA and PsA in our study demonstrate at least one classical symptom of OSA. Furthermore, snoring was reported by 95% of OSA patients with PsA. Nonetheless, EDS was reported by 29% of participants only, which aligns well with previous reports.^[12,32]^ Dejcman et al reported that only 47.6% of patients with PsA and OSA experienced EDS, all of whom had severe OSA.^[12]^ Dejcman et al also concluded that EDS (ESS score ≥ 10) was not predictive of OSA (p ≈ 0.37).^[12]^ Petzinna et al found that 17.6% of patients with PsA experienced EDS, none of the patients diagnosed with OSA exhibited excessive sleepiness, and no significant relationship was found between EDS and OSA in patients with PsA.^[32]^ One possible explanation for the low frequency of EDS despite high frequency of snoring in participants of the current study could be the effect of chronic pain and inflammation in patients with PsA. This may alter the perception or reporting of sleepiness in this group of patients leading to underestimation of EDS. This hypothesis, however, warrants further exploration, as it may provide insight into the unique manifestations of OSA in patients with PsA. On a similar note, we observed no associations between comorbidities and OSA in patients with PsA. Whether patients with PsA (and possibly other related autoimmune diseases) are experiencing a distinct clinical phenotype or a pathophysiological endotype of OSA characterized by low levels of sleepiness and commodities merits further investigation.

Moreover, a significantly higher proportion of female participants exhibited moderate-to-severe disease activity, compared to those in remission or with low disease activity (83% vs 56%, *P* = .049). This aligns with existing literature,^[34,35]^ Lubrano et al^[34]^ reported that female patients with PsA had higher disease activity score (based on DAPSA), more tender and swollen joints, greater functional limitations, higher disease burden and a lower rate of remission compared to males.^[34]^ Surprisingly, the current study identified no significant correlation between PsA disease activity and the occurrence of OSA. This aligns with the findings of Petzinna et al^[32]^ who also reported no relationship between PsA disease activity (assessed by Disease Activity Score-28, (DAS28)) and OSA prevalence.^[32]^ In contrast, a Danish nationwide cohort study encompassing approximately 40,000 individuals who had been followed for 15 years, showed a bidirectional relationship between PsA and OSA.^[11]^ Patients with PsA experienced a 1.75-fold increase in incident rate ratio for developing OSA compared to the general population.^[11]^ This discrepancy may be explained by the fact that OSA is multifaceted, and its link with PsA could be influenced by factors other than PsA disease activity. For example, management of PsA with immunomodulators may have potent therapeutic effect on systemic inflammation, potentially reducing the likelihood of detecting a direct relationship between OSA and PsA. Additionally, our study included a relatively small sample size, which may limit its statistical power, hindering the detection of subtle associations between these conditions.

Unlike earlier studies reporting no significant predictors for OSA in patients with PsA,^[11,12,32]^ our study demonstrates that increasing age, as well as greater BMI which showed a trend toward significance, were predictors of OSA in this population. This finding aligns with trends observed in the general population, where the risk of OSA increases with age and higher BMI. This primarily may be due to anatomical changes such as the enlargement of pharyngeal soft tissues, reduced muscle tone, and the weakening of airway structures, all of which can contribute to airway collapse during sleep.^[10,36]^ However, it is unknown whether PsA patients are experiencing additional mechanisms for airway narrowing and obstruction that is distinct to patients with OSA and without PsA. Further research is also required to scrutinize this interlacing relationship.

### 4.1. Limitations and Strengths

Although the findings from the current study are novel, several limitations can be acknowledged. First, we used a cross-sectional research design in the current study. Although this design is particularly suitable for estimating prevalence, it precludes establishing causality or determining temporal relationship between PsA and OSA. Nonetheless, we used gold-standard PSG for diagnosing OSA, which strengthens the reliability and validity of our findings. Our study also included a relatively small sample size which may reduce statistical power, potentially limiting the ability to detect subtle associations between PsA and OSA. Additionally, a significant proportion (38%) of potential participants declined to undergo PSG. Although this may introduce selection bias, nonparticipation rate is a common challenge in PSG-based research due to the cumbersome nature of the procedure. It is possible that patients with more pronounced sleep-related symptoms were more likely to accept participation in the current study, which could have led to an overestimation of OSA prevalence in the broader PsA population. However, as both groups had comparable ESS scores; and thus, similar levels of EDS, it is unlikely that this bias substantially affected the findings. Moreover, the clinical burden of PsA itself may contribute to higher nonparticipation rates, particularly among those with greater disease activity. For this reason, we are more concerned that the prevalence reported here may underestimate, rather than overestimate, the true prevalence of OSA in patients with PsA. Nevertheless, we believe that our findings remain informative given the rarity of PsA among visitors to Rheumatology clinics, and the substantial impact of the disease on patient health, leading to greater nonparticipation rate in experimental studies like ours. This study was also conducted in a single tertiary care center, limiting the generalizability of our findings to other populations, and findings should be interpreted with caution. Finally, we acknowledge that the impact of PsA treatment was not thoroughly examined in this study, leaving their potential influence on OSA prevalence unexplored. Treatment is an important potential confounder; however, the relatively small sample size in the current study limited full consideration of its effects. Nevertheless, the shared relationship between OSA and PsA pathophysiology through inflammatory cytokines remains an important mechanistic link between the 2 conditions. This is beyond the scope of the current study, but indeed, requires careful consideration in future analyses.^[5,11,14,15,19–22]^ Additionally, the study did not evaluate skin lesions or specific types of psoriasis, which may be linked with OSA. Future studies with larger sample sizes; multi-central, longitudinal designs; and comprehensive data on PsA treatments and comorbidities are needed to further clarify the relationship between PsA and OSA.

## 5. Conclusion

Our study highlights a higher prevalence of OSA among patients with PsA (50%) compared to the population in the same region. However, no significant correlation was found between PsA disease activity and the presence of OSA. Although tentative, this finding is interesting, and may be explained by the influence of anti-inflammatory therapies on PsA activity which may potentially mask any conceivable association. Our findings support maintaining high suspicion index and early screening for OSA when providing clinical care for patients with PsA, especially those with obesity or old age. Future research with larger sample sizes, longitudinal designs, and a detailed assessment of the impact of PsA treatments on OSA are needed to untangle the complex relationship between PsA and OSA.

## Acknowledgments

The authors would like to thank Prof Sami Bahlas, and Dr Roaa Alsulaimani from the Rheumatology Division, Department of Medicine, King Abdulaziz University, Jeddah, Saudi Arabia for their contribution in this study.

## Author contributions

**Conceptualization:** Mohammad Mustafa, Suzan Attar, Omar Kanbr, Yasser Bawazir, Ahmad A. Bamagoos, Ayah Boudal, Faris Alhejaili, Siraj Wali.

**Data curation:** Mohammad Mustafa, Suzan Attar, Omar Kanbr, Yasser Bawazir, Ahmad A. Bamagoos, Ayah Boudal, Faris Alhejaili, Siraj Wali.

**Formal analysis:** Ahmad A. Bamagoos, Siraj Wali.

**Funding acquisition:** Siraj Wali.

**Investigation:** Mohammad Mustafa, Suzan Attar, Yasser Bawazir, Ahmad A. Bamagoos, Siraj Wali.

**Methodology:** Mohammad Mustafa, Suzan Attar, Omar Kanbr, Yasser Bawazir, Ahmad A. Bamagoos, Siraj Wali.

**Project administration:** Siraj Wali.

**Supervision:** Siraj Wali.

**Writing – original draft:** Mohammad Mustafa, Suzan Attar, Omar Kanbr, Yasser Bawazir, Ahmad A. Bamagoos, Ayah Boudal, Faris Alhejaili, Siraj Wali.

**Writing – review & editing:** Mohammad Mustafa, Suzan Attar, Yasser Bawazir, Ahmad A. Bamagoos, Ayah Boudal, Faris Alhejaili, Siraj Wali.
